# Cell-based analysis of *CAD* variants identifies individuals likely to benefit from uridine therapy

**DOI:** 10.1038/s41436-020-0833-2

**Published:** 2020-05-28

**Authors:** Francisco del Caño-Ochoa, Bobby G. Ng, Malak Abedalthagafi, Mohammed Almannai, Ronald D. Cohn, Gregory Costain, Orly Elpeleg, Henry Houlden, Ehsan Ghayoor Karimiani, Pengfei Liu, M. Chiara Manzini, Reza Maroofian, Michael Muriello, Ali Al-Otaibi, Hema Patel, Edvardson Shimon, V. Reid Sutton, Mehran Beiraghi Toosi, Lynne A. Wolfe, Jill A. Rosenfeld, Hudson H. Freeze, Santiago Ramón-Maiques

**Affiliations:** 1 Genome Dynamics and Function Program, Centro de Biología Molecular Severo Ochoa, CSIC-UAM, Madrid, Spain; 2 Group 739, Centro de Investigación Biomédica en Red de Enfermedades Raras (CIBERER)–Instituto de Salud Carlos III, Valencia, Spain; 3 Human Genetics Program, Sanford Burnham Prebys Medical Discovery Institute, La Jolla, CA USA; 4 Genomics Research Department, Saudi Human Genome Project, King Fahad Medical City and King Abdulaziz City for Science and Technology, Riyadh, Saudi Arabia; 5 Section of Medical Genetics, Children’s Hospital, King Fahad Medical City, Riyadh, Saudi Arabia; 6 Division of Clinical and Metabolic Genetics, The Hospital for Sick Children, Toronto, ON Canada; 7 Division of Paediatric Medicine, The Hospital for Sick Children, Toronto, ON Canada; 8 Department of Paediatrics, University of Toronto, Toronto, ON Canada; 9 Department of Molecular Genetics, University of Toronto, Toronto, ON Canada; 10 Centre for Genetic Medicine, The Hospital for Sick Children, Toronto, ON Canada; 11 Department of Genetics, Hadassah-Hebrew University Medical Center, Jerusalem, Israel; 12 Department of Neuromuscular disorders, UCL Institute of Neurology University College, London, UK; 13 Molecular and Clinical Sciences Institute, St. George’s, University of London, Cranmer Terrace, London, UK; 14 Department of Molecular and Human Genetics, Baylor College of Medicine, Houston, TX USA; 15 Baylor Genetics Laboratories, Houston, TX USA; 16 Department of Neuroscience and Cell Biology and Child Health Institute of New Jersey, Rutgers Robert Wood Johnson Medical School, New Brunswick, NJ USA; 17 Department of Pediatrics/Division of Genetics, Medical College of Wisconsin, Milwaukee, WI USA; 18 Genomic Science and Precision Medicine Center, Medical College of Wisconsin, Milwaukee, WI USA; 19 Department of Pediatric Neurology, National Neuroscience Institute, King Fahad Medical City, Riyadh, Saudi Arabia; 20 Department of Neurology (Section of pediatric neurology) Children’s Hospital of Wisconsin, Medical of College of Wisconsin, Milwaukee, WI USA; 21 Pediatric Neurology Unit, Hadassah-Hebrew University Medical Center, Jerusalem, Israel; 22 Department of Molecular, Human Genetics Baylor College of Medicine & Texas Children’s Hospital, Houston, TX USA; 23 Department of Pediatric Diseases, Faculty of Medicine, Mashhad University of Medical Sciences, Mashhad, Iran; 24 Undiagnosed Diseases Program, Common Fund, National Institutes of Health, Bethesda, MD USA; 25 Present Address: Instituto de Biomedicina de Valencia (IBV-CSIC), Valencia, Spain

**Keywords:** congenital disorder of glycosylation, de novo pyrimidine biosynthesis, carbamoyl phosphate synthetase, aspartate transcarbamoylase, dihydroorotase

## Abstract

**Purpose:**

Pathogenic autosomal recessive variants in *CAD*, encoding the multienzymatic protein initiating pyrimidine de novo biosynthesis, cause a severe inborn metabolic disorder treatable with a dietary supplement of uridine. This condition is difficult to diagnose given the large size of *CAD* with over 1000 missense variants and the nonspecific clinical presentation. We aimed to develop a reliable and discerning assay to assess the pathogenicity of *CAD* variants and to select affected individuals that might benefit from uridine therapy.

**Methods:**

Using CRISPR/Cas9, we generated a human *CAD*-knockout cell line that requires uridine supplements for survival. Transient transfection of the knockout cells with recombinant *CAD* restores growth in absence of uridine. This system determines missense variants that inactivate CAD and do not rescue the growth phenotype.

**Results:**

We identified 25 individuals with biallelic variants in *CAD* and a phenotype consistent with a CAD deficit. We used the *CAD*-knockout complementation assay to test a total of 34 variants, identifying 16 as deleterious for CAD activity. Combination of these pathogenic variants confirmed 11 subjects with a CAD deficit, for whom we describe the clinical phenotype.

**Conclusions:**

We designed a cell-based assay to test the pathogenicity of *CAD* variants, identifying 11 CAD-deficient individuals who could benefit from uridine therapy.

## INTRODUCTION

**Fig. 1:**
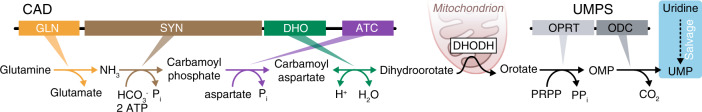
Schematic of the pathway for de novo biosynthesis of the pyrimidine nucleotide uridine 5-monophosphate (UMP). The initial enzymatic activities, glutaminase (GLN), carbamoyl phosphate synthetase (SYN), aspartate transcarbamoylase (ATC), and dihydroorotase (DHO) are fused into the multifunctional protein CAD. The next reaction after CAD is catalyzed by dihydroorotate dehydrogenase (DHODH), an enzyme anchored to the inner mitochondrial membrane. The last two steps are catalyzed by UMP synthase (UMPS), a bifunctional enzyme with orotate phosphoribosyl transferase (OPRT) and orotidine decarboxylase (ODC) activities. Alternatively, UMP can be obtained from uridine through salvage pathways (depicted in cyan).

*CAD* encodes a multienzymatic cytoplasmic protein harboring four functional domains, each catalyzing one of the initial reactions for de novo biosynthesis of pyrimidine nucleotides: glutamine amidotransferase (GLN), carbamoyl phosphate synthetase (SYN), aspartate transcarbamoylase (ATC), and dihydroorotase (DHO)^1–3^ (Fig. 1). This metabolic pathway is essential for nucleotide homeostasis, cell growth, and proliferation.^4^ Defects in dihydroorotate dehydrogenase (DHODH) or UMP synthetase (UMPS), the enzymes catalyzing the next steps in the pathway after CAD, are associated with severe human disorders (Miller syndrome [OMIM 263750]^5^ and orotic aciduria [OMIM 258900]^6^). In 2015, we identified a single individual with early infantile epileptic encephalopathy and two variants in *CAD*, one an in-frame deletion of an exon and the other a missense variant (p.R2024Q) in a highly conserved residue.^7^ Metabolic analysis of subject fibroblasts showed impaired CAD activity–dependent incorporation of ^3^H-labeled aspartate into nucleic acids and nucleotide sugars, precursors for glycoprotein synthesis. Uridine supplements corrected this CAD-associated congenital disorder of glycosylation (CDG; OMIM 616457), suggesting a simple potential treatment. In two subsequent reports, five affected individuals from four unrelated families with similar symptoms showed likely pathogenic variants in *CAD*, but no functional studies were done.^8,9^ However, uridine treatment of three suspected individuals showed striking improvement, with cessation of seizures and significant progression from minimally conscious state to communication and walking. Recently, uridine triacetate (Xuriden) was approved by the FDA to treat hereditary orotic aciduria;^10^ presumably, it could be used to treat affected individuals with CAD deficiency.

The attractiveness of a simple therapy brought 25 suspected individuals to our attention for evaluation. Unfortunately, the metabolic labeling assay using ^3^H-labeled aspartate has a low resolution and a narrow dynamic range. To have a more reliable and discerning assay, we tested the ability of each variant to rescue growth of a human *CAD*-knockout cell line that requires uridine supplements for survival. Surprisingly, only 11 of 25 suspected individuals had pathologic variants and would potentially benefit from uridine supplements. We describe the development of this functional assay, the general clinical phenotype, and analysis of these individuals. We caution about relying on current prediction programs to assess pathogenicity of variants for this large multifunctional enzyme.

## MATERIALS AND METHODS

### Clinical data

Informed consent was provided by all subjects in accordance with each clinician’s individual institution. Additional consent to analyze samples was provided in accordance with Sanford Burnham Prebys Medical Discovery Institute (IRB-2014-038-17).

### CRISPR/Cas9 plasmid

pSpCas9 (BB)-2A-Puro (PX459) vector (Addgene), encoding Cas9, was digested with BbsI and purified with Qiaquick Gel Extraction kit (Qiagen). Complementary double-stranded DNA (dsDNA) oligonucleotides encoding single guide RNA (sgRNA), designed to target the first exon of *CAD*, were purchased (Sigma) with 5’ overhangs complementary to the BbsI site and an extra G base to favor transcription^11^ (Table S1). The oligonucleotides were phosphorylated with T4 polynucleotide kinase (NEB), annealed, and inserted in the linearized vector with T4 DNA ligase (NEB). The construct was amplified in TOP10 *E. coli* cells (ThermoFisher), verified by sequencing, and purified with a Plasmid Midi kit (Qiagen).

### GFP-CAD plasmid

Enhanced green fluorescent protein (GFP) coding sequence was obtained by HindIII and KpnI digestion of pPEU2 vector (kindly provided by Dr. Nick Berrow, IRB Barcelona), and ligated into pCDNA3.1 (Promega) linearized with same restrictions enzymes. The resulting plasmid (pcDNA3.1-GFP) was verified by sequencing. Human *CAD* was polymerase chain reaction (PCR) amplified from complementary DNA (cDNA) (Open Biosystems clone ID 5551082) using specific primers (Table S1) and ligated with In-Fusion (Clontech) into NotI linearized pcDNA3.1-GFP. The resulting plasmid (pcDNA3.1-GFPhuCAD) encodes an N-terminal histidine-tagged GFP followed in-frame by human CAD. Site-directed mutagenesis was carried out following the QuickChange protocol (Stratagene) and a pair of specific oligonucleotides (Table S1) and PfuUltra High-Fidelity DNA polymerase (Agilent).

### Generating a *CAD*-knockout cell line

Human U2OS (bone osteosarcoma) cells were grown in DMEM (Lonza), 10% fetal bovine serum (FBS; Sigma), 2 mM L-glutamine (Lonza), and 50 U·ml^−1^ penicillin and 50 μg·ml^−1^ streptomycin (Invitrogen), at 5% CO_2_ and 37 °C. One day before transfection, 1.5–2 × 10^5^ U2OS cells in a final volume of 500 µl of medium were transferred to 24-well plates to reach approximately 50–80% confluence. For transfection, 2 µg of DNA in 50 µl of DMEM and 50 µl of FuGene6 transfection reagent (Promega) at 1 mg·ml^−1^ in DMEM were incubated separately for 5 minutes at room temperature, and then mixed together and incubated at room temperature for an additional 10 minutes. The 100 µl mix was added to the wells drop by drop, followed by a 16-hour incubation at 37 °C and 5% CO_2_. Twenty-four hours post transfection, puromycin was added for one week to select transfected cells and enhance Cas9 cleavage. Media was supplemented with 30 µM uridine (Sigma) to allow growth of CAD-deficient cells. Individual cells were isolated by serial dilution in 96-well plates, seeded into 24-well plates, and expanded for 2–3 weeks. To identify CAD-deficient clones, a replica of the plate was grown in media with 10% fetal bovine macroserum (FBM) without uridine, instead of FBS. FBM was prepared as reported.^12^ In brief, 50 ml of heat-inactivated FBS were dialyzed against 1 L of tap water for 1 day at 4 °C using SpectraPor #3 dialysis tubing with a molecular weight cutoff of 3500 Da (Spectrum Laboratories, Inc., USA), supplemented with NaCl (9 g per liter), sterilized with a 0.22-µm filter, and stored at −20 °C. Disruption of *CAD* was confirmed by Sanger sequencing. For this, exon 1 of *CAD* was PCR amplified with specific primers (Table S1), inserted in ZeroBlunt vector (Invitrogen) and sequenced with M13 primer. CAD-deficient cells were confirmed by western blot and immunofluorescence microscopy using a monoclonal antibody (Cell Signaling Technology, #93925).

### Growth complementation assay

U2OS *CAD*-KO cells were transfected with wild-type (WT) or mutated pcDNA3.1-GFPhuCAD using FuGene6 as detailed above. One day after transfection, 1 × 10^5^ cells were seeded by duplicate in 24-well plates using media supplemented with 10% FBM (without uridine). Every 24 hours, cells from one well were trypsinized and counted using a Countess II FL Automated Cell Counter (Thermo) or a Neubauer chamber. Doubling time was calculated using an online tool (http://www.doubling-time.com/compute.php).

## RESULTS

### Validation of a growth complementation assay in *CAD*-knockout cells

**Fig. 2:**
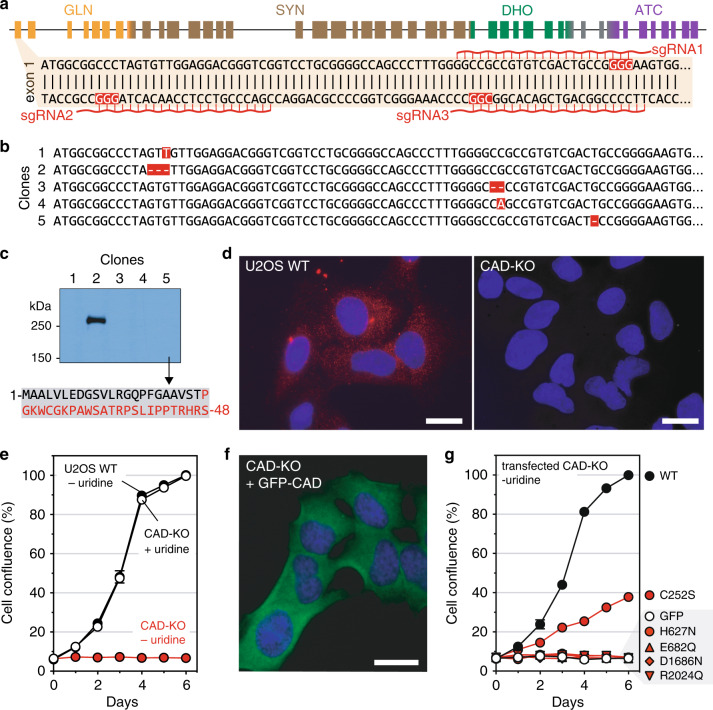
Using CRISPR/Cas9 to knockout *CAD* in U2OS cells. (**a**) Schematic representation of *CAD* locus, with 44 exons colored according to their respective functional domains; detail of the 5’ region of exon 1, indicating the single guide RNA (sgRNA) with protospacer adjacent motif (PAM) sequences in red boxes. (**b**) Sequencing of five clones selected after CRISPR-Cas9 editing shows insertions and deletions (highlighted in red) in exon 1. (**c**) Expression of CAD in total lysates of clones shown in (**a**) analyzed by western blot with a monoclonal antibody. Clone 5, chosen as the *CAD*-knockout (KO) cell for further studies, produces an early truncated CAD protein of 48 residues with an incorrect sequence colored in red. (**d**) Immunofluorescence of wild-type (WT) and *CAD*-KO U2OS cells, using a monoclonal antibody against CAD (red signal) and nuclear labeling with Hoechst (blue signal). (**e**) Proliferation assay of *CAD*-KO cells in media with or without uridine, compared with the growth of WT cells. (**f**) Imaging of *CAD*-KO cells transiently transfected with GFP-CAD, using GFP fluorescent signal (green) and Hoechst (blue). (**g**) Transfection of GFP-CAD rescues the growth phenotype of *CAD*-KO in uridine-deprived media. Cells transfected with GFP alone do not proliferate. Cells transfected with GFP-CAD variants bearing well-characterized inactivating variants in the SYN, DHO, or ATC domains fail to proliferate without uridine, whereas the inactivation of the GLN domain (variant C252S) allows limited growth. Scale bars in (**d**, **f**) indicate 20 µm.

We wanted to create a *CAD*-knockout (KO) cell line that could be used to assess the pathogenicity of *CAD* variants. Using CRISPR/Cas9 technology, we knocked out *CAD* in human U2OS cells by selecting an isogenic clone that introduced a homozygous c.70delG frameshift (p.Ala24Profs*27) within exon 1 (Fig. 2a–c). We verified by western blot and immunofluorescence that *CAD*-KO cells do not express CAD (Fig. 2c, d). As expected, these cells are unable to grow in absence of uridine, but proliferate at similar rate as WT cells in media supplemented with 30 µM exogenous uridine (Fig. 2e). Next, we transiently transfected KO cells with a plasmid encoding human CAD fused to the enhanced GFP at the N-terminus (Fig. 2f). *CAD*-KO cells expressing GFP-CAD proliferated in uridine-deprived conditions at a normal rate (doubling time ∼1 day), whereas cells transfected with GFP alone did not grow (Fig. 2g).

To confirm that all four enzymatic activities of CAD were needed for de novo pyrimidine synthesis and cell growth in absence of uridine, we measured the proliferation of *CAD*-KO cells transfected with GFP-CAD bearing well-known inactivating variants for each activity (Fig. 2g). The transfected inactivated variants in the SYN (p.H627N, p.E682Q),^13,14^ DHO (p.D1686N),^15^ and ATC (p.R2024Q)^7,16^ domains failed to rescue the growth of *CAD*-KO cells. In turn, the GLN inactive mutant (p.C252S)^17^ showed a partial rescue, with transfected cells doubling every ∼2.5 days, suggesting that free ammonia can, to some extent, contribute to the synthesis of carbamoyl phosphate (Fig. 1).

### Identification and impact of potential CAD variants

Since *CAD* encodes a large protein with 2225 amino acids covering 44 exons (Fig. 2a), it is not surprising that all previously reported (*n* = 6) affected individuals were identified using next-generation sequencing (NGS).^7–9^ Likewise, using NGS we identified 25 potential CAD-deficient individuals based on the presence of biallelic variants and a clinical phenotype similar to previously reported individuals (Table 1). Ultimately, we tested 34 variants of uncertain significance (VUS) in our validated knockout assay.

**Table 1:** Summary of *CAD* variants.

ID^a^	cDNA^b^	Amino acid	SIFT category	SIFT value^c^	PolyPhen2 category	PolyPhen2 value^c^	CADD PHRED^c^	KO rescue results	gnomAD carriers/alleles
Baylor - 001^d^	c.2156+5G>A	NA	NA	NA	NA	NA	10.62	NA	4/251138
	c.4667A>C	p.K1556T	tolerated	0.38	possibly_damaging	0.631	24.1	Pathogenic	1/251278
Baylor - 002	c.5147C>T	p.T1716M	deleterious	0	probably_damaging	0.982	25.9	Benign	17/281494
	c.5561G>A	p.R1854Q	tolerated	0.25	benign	0.186	23.8	Benign	15/282772
Baylor - 003	c.2372A>C	p.D791A	deleterious	0.04	possibly_damaging	0.473	26.8	Benign	NA
	c.4487G>C	p.G1496A	deleterious	0	probably_damaging	0.999	27	Benign	NA
Baylor - 004	c.713G>A	p.R238H	deleterious	0.05	benign	0.104	18.27	Benign	109/282876
	c.1159G>A	p.G387S	tolerated	0.67	benign	0	7.152	Benign	11/251452
Baylor - 005	c.4501T>A	p.C1501S	tolerated	0.44	possibly_damaging	0.636	23.8	Benign	1/251356
	c.6556C>T	p.P2186S	deleterious	0	probably_damaging	0.999	32	Pathogenic	NA
Baylor - 006	c.419A>G	p.Q140R	tolerated	0.42	benign	0.029	18.14	Benign	2/282842
	c.5570G>A	p.R1857Q	tolerated	0.17	benign	0.022	23.7	Benign	8/282788
Baylor - 007	c.943G>A	p.A315T	deleterious	0.01	probably_damaging	0.971	26.4	Benign	4/251364
	c.5353C>T	p.R1785C	deleterious	0	probably_damaging	0.994	28.5	Pathogenic	3/251030
Baylor - 008	c.785T>C	p.I262T	tolerated	0.15	benign	0.185	22.7	Benign	7/282792
	c.3868G>A	p.G1290S	tolerated	0.07	benign	0.058	16.78	Benign	31/282488
Baylor - 009	c.5147C>T	p.T1716M	deleterious	0	probably_damaging	0.982	25.9	Benign	17/281494
	c.5561G>A	p.R1854Q	tolerated	0.25	benign	0.186	23.8	Benign	15/282772
Baylor - 010	c.3649G>A	p.V1217I	deleterious	0.02	possibly_damaging	0.483	25	Benign	3/251356
	c.4568C>T	p.A1523V	tolerated	0.05	benign	0.391	23.6	Benign	NA
Baylor - 011	c.959A>G	p.N320S	deleterious	0.02	benign	0.077	22.4	Pathogenic	9/282728
	c.2984C>G	p.S995C	deleterious	0	probably_damaging	0.995	31	Benign	NA
**CDG - 0017**	c.1576G>A	p.G526R	deleterious	0	possibly_damaging	0.657	26.7	Pathogenic	4/251224
	c.1576G>A	p.G526R	deleterious	0	possibly_damaging	0.657	26.7	Pathogenic	4/251224
**CDG - 0104**	c.5959C>G	p.L1987V	deleterious	0	probably_damaging	0.992	26.4	Pathogenic	NA
	c.5959C>G	p.L1987V	deleterious	0	probably_damaging	0.992	26.4	Pathogenic	NA
**CDG - 0105**	c.5959C>G	p.L1987V	deleterious	0	probably_damaging	0.992	26.4	Pathogenic	NA
	c.5959C>G	p.L1987V	deleterious	0	probably_damaging	0.992	26.4	Pathogenic	NA
**CDG - 0111**	c.6329G>T	p.R2110L	tolerated	0.2	Benign	0.046	16.83	Pathogenic	1/251314
	c.6329G>T	p.R2110L	tolerated	0.2	Benign	0.046	16.83	Pathogenic	1/251314
**CDG - 0112**	c.3098G>A	p.R1033Q	deleterious	0.01	possibly_damaging	0.537	31	Pathogenic	7/251346
	c.3098G>A	p.R1033Q	deleterious	0.01	possibly_damaging	0.537	31	Pathogenic	7/251346
**CDG - 0117**	c.5957G>A	p.R1986Q	deleterious	0.01	probably_damaging	0.992	33	Pathogenic	3/249932
	c.5957G>A	p.R1986Q	deleterious	0.01	probably_damaging	0.992	33	Pathogenic	3/249932
**CDG - 0118**	c.6382G>A	p.E2128K	tolerated	0.15	possibly_damaging	0.578	26.2	Pathogenic	NA
	c.6382G>A	p.E2128K	tolerated	0.15	possibly_damaging	0.578	26.2	Pathogenic	NA
**CDG - 0122**	c.3512C>A	p.P1171Q	deleterious	0	probably_damaging	0.936	28.4	Pathogenic	1/251476
	c.4315-1G>A	NA	NA	NA	NA	NA	34	NA	1/31408
**CDG - 0123**	c.2995G>A	p.V999M	deleterious	0	probably_damaging	1	29.8	Pathogenic	NA
	c.2995G>A	p.V999M	deleterious	0	probably_damaging	1	29.8	Pathogenic	NA
**CDG - 0278**	c.98T>G	p.M33R	deleterious	0	benign	0.223	24.9	Pathogenic	1/243848
	c.98T>G	p.M33R	deleterious	0	benign	0.223	24.9	Pathogenic	1/243848
CDG - 0443	c.713G>A	p.R238H	deleterious	0.05	benign	0.104	18.27	Benign	109/282876
	Uniparental disomy Chr. 2								
CDG - 1000	c.4669C>G	p.L1557V	tolerated	0.13	benign	0.003	21.9	Benign	62/282658
	c.6320C>G	p.P2107R	tolerated	0.15	benign	0	16.49	Benign	2/282698
CDG - 1001	c.2386C>A	p.P796T	tolerated	0.53	benign	0.039	21.1	Pathogenic	10/282430
	c.4735G>A	p.E1579K	tolerated	0.26	possibly_damaging	0.624	23.7	Benign	5/250930
**CDG - 1046**	c.887G>A	p.G296E	deleterious	0	probably_damaging	1	26.4	Pathogenic	6/251446
	c.2225G>A	p.R742Q	deleterious	0.03	benign	0.414	25.3	Pathogenic	NA

*cDNA* complementary DNA, *KO* knockout.

^a^CAD-deficient subjects are denoted with ID in bold.

^b^cDNA (NM_004341.5), Uniprot ID (P27708).

^c^SIFT Value (Closer to 0 is damaging), Polyphen (Closer to 1 is damaging), CADD (20 puts variant in top 1% of deleterious variants, 30 in top 0.1%).

^d^This individual was found to have both variants in *cis*.

**Fig. 3:**
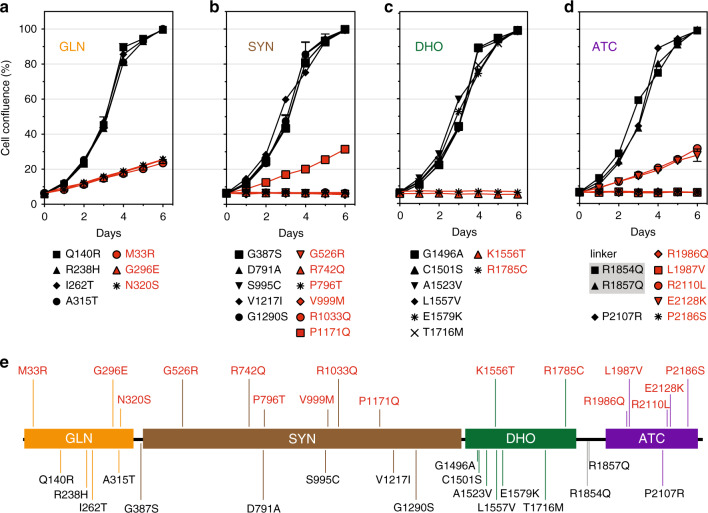
Assessing the pathogenicity of *CAD* variants. (**a–d**) Growth complementation assay of *CAD*-knockout (KO) cells grown in absence of uridine and transfected with GFP-CAD bearing point variants in the GLN (**a**), SYN (**b**), DHO (**c**), or ATC (**d**) domains. Variants in the loop connecting the DHO and ATC domains are included in (**d**). Cell proliferation is represented as % confluence with respect to cells transfected with GFP-CAD wild type (WT). Each point represents the mean and standard deviation of three measurements, and all mutants were tested in at least two independent experiments. Variants compromising CAD activity are colored in red. (**e**) Linear representation of CAD, mapping the inactivating (in red) and benign (in black) variants.

To assess the damaging potential of variants found in subjects, we transfected *CAD*-KO cells with GFP-CAD bearing the clinical variants and monitored proliferation in uridine-deprived conditions (Fig. 3a–d). Each newly constructed plasmid carrying an individual-specific variant required complete sequencing of the ∼8 kb *GFP-CAD* cDNA to ensure no additional changes were introduced during PCR. We also verified the efficiency of the transfection (>95%) and that the mutated proteins were being expressed by imaging the GFP fluorescence signal in the *CAD*-KO cells two days after transfection (data not shown).

Three of the seven variants found in the GLN domain, p.M33R, p.G296E, and p.N320S, showed a partial rescue (Fig. 3a). The doubling time was similar to the cells transfected with the GLN inactivating variant p.C252S (Fig. 2g), indicating that these variants impair the GLN domain. On the other hand, cells transfected with SYN variants p.G526R, p.R742Q, p.P796T, p.V999M, and p.R1033Q failed to proliferate, whereas the variant p.P1171Q showed a partial rescue (Fig. 3b). Of the eight variants of the DHO domain tested, only two, p.K1556T and p.R1785C, failed to restore cell growth (Fig. 3c). For the ATC variants, three variants, p.R1986Q, p.L1987V and p.P2186S, failed to rescue the cells, whereas p.R2110L and p.E2128K allowed a partial rescue (Fig. 3d). Finally, transfection with the two variants found at the linker between the DHO and ATC domains (p.R1854Q and p.R1857Q) restored normal growth (Fig. 3d).

Based on these results, we concluded that the failure to rescue the growth phenotype of *CAD*-KO cells in absence of uridine indicates that 16 of the 34 variants tested have a deleterious effect on CAD activity and therefore are pathogenic.

Interestingly, significant differences were seen when comparing the results of the KO assay with three popular in silico prediction programs (SIFT,^18^ PolyPhen-2,^19^ CADD^20^) (Table 1). All three prediction programs agreed with each other for 20/34 variants (59%; 15/34 pathogenic, 5/34 benign variants). Yet only 38% (13/34) (9/34 pathogenic, 4/34 benign) of the variants agreed in all three prediction programs and the complementation assay. We used a CADD PHRED score of above 20, which places a variant in the top 1% deleterious variants in the human genome as potentially pathogenic. Below 20, we considered likely benign.

The mechanisms of inactivation of the pathogenic variants will be described in a separate study.

### Clinical

To date, only six affected individuals from five unrelated families have been identified with CAD deficiencies.^7–9^ The clinical presentation is general in nature, but all these individuals showed varying severities of neurological involvement including developmental delays and/or seizures. Furthermore, all had hematological abnormalities including abnormal red blood cells (anisopoikilocytosis) and anemia. Two of the six are reported to be deceased, while the remaining four received uridine.

In this study, we identified 25 individuals with biallelic variants in *CAD*, who presented with a phenotype potentially consistent with CAD deficiency. We used the *CAD*-KO complementation assay described above to determine the pathogenicity of each variant identified and ultimately confirmed 11 CAD-deficient subjects (Table 1, Fig. 3e).

**Fig. 4:**
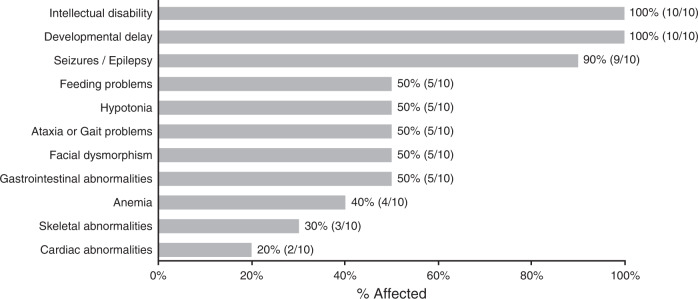
Clinical summary for ten unreported CAD-deficient individuals. Clinical information for ten of the available subjects was collected and summarized as % affected.

Detailed clinical information was available and provided for 10 of the 11 confirmed individuals (Fig. 4). Consistent with the initial CAD-deficient individuals,^7,8^ all ten individuals presented here showed varying neurological abnormalities. All had intellectual and developmental disability, while 9/10 (90%) had seizure activity. Gastrointestinal complications ranging from feeding problems, reflux, and recurrent vomiting were seen in half (5/10) of the individuals, as was facial dysmorphism, hypotonia, and ataxia. All five of the previously identified subjects showed hematological abnormalities, while in our cohort this was 4/10 (40%). Less affected systems included the skeleton (3/10) and the heart (2/10).

In our cohort, one individual was noted to have died (CDG-0118). However, four families (0017, 0104, 0118, 0123) were noted to have a family history of multiple affected siblings with a similar presentation. From these four families, three had at least one sibling with a similar disorder who expired.

Due to the lack of detailed clinical information, CDG-0117 was not included in the final summary. However, he was noted to have structural brain abnormalities and a family history significant for premature death in two affected female siblings. Importantly, genomic DNA was available for one of the two deceased siblings and was found to also carry the same homozygous c.5957G>A [p.R1986Q] *CAD* variant.

One family (CDG-0112) had a dual diagnosis of CAD deficiency and a recessive intellectual developmental disorder with cardiac arrhythmia (OMIM 617173). Within this family, both affected siblings harbored a homozygous pathogenic c.249+3A>G [p.Asp84Valfs31*] variant in *GNB5*,^21^ but only the male sibling carried the pathogenic homozygous c.3098G>A [p.R1033Q] variant in *CAD*. Given the clinical similarities of these two disorders, especially the neurological features, we cannot determine which symptoms are due to specifically the CAD variant alone.

## DISCUSSION

The prospect of a simple, nontoxic therapy for a potentially lethal disorder excites all stakeholders: patients, caretakers, physicians, and scientists. Identifying the first CAD-deficient individual and showing that uridine corrects cellular defects set the stage for the highly successful use of uridine in two CAD-deficient individuals.^7,8^ As a result, and given the nonspecific clinical presentation of CAD-deficient individuals, we received many requests to test subject fibroblasts in a functional assay that involves labeling cells with ^3^H-aspartate to measure the CAD-dependent contribution to de novo pyrimidine synthesis (Fig. 1). However, the assay has a limited dynamic range (~2-fold) and many determinations left us ambivalent and uncertain about the diagnosis. Thus, a new robust and reliable biochemical assay was required to evaluate the pathogenicity of *CAD* variants.

We designed a *CAD*-knockout cell line whose growth was dependent on added uridine (Fig. 2) and then tested each variant for its ability to rescue uridine-independent growth (Fig. 3a–d). Most of the variants either fully rescued growth, meaning the variants were benign, or were unable to rescue growth completely, showing they were pathologic variants. Only a few showed partial rescue, which we interpret to mean a damaging variant that decreases, but does not eliminate the activity. When each variant was combined based on individual-specific genotyping, we determined which individuals indeed had a CAD deficiency and therefore predict which ones would benefit from uridine therapy (Fig. 3e and Table 1). This is a stringent prediction based on each single variant. It does not test the specific combination of alleles found in each individual, but we assume the combination of two variants would not cancel each other to generate a fully capable CAD protein. If this were the case, it is unlikely that the individuals themselves would show the expected clinical phenotype. Surprisingly only 11 of the 25 suspected individuals appeared to be authentic cases based on this functional assay.

We also compared our assay results with three prediction programs designed to assess the pathogenicity of each variant (Table 1). There was considerable disagreement between the programs for many variants, and the programs produced both false positive and false negative results. Based on these findings, we suggest that any suspected CAD cases first be validated using this (or similar) biochemical assay. And it is likely that more putative CAD-deficient cases will be suspected, since *CAD* has ~1020 missense rare variants in the public gnomAD browser (Ver2.1.1) database (https://gnomad.broadinstitute.org; accessed 23 January 2020 with 125,748 exomes and 15,708 genomes). Some families may choose to start uridine therapy without benefit of these results. That is certainly possible since the uridine is available to families and subjects over the Internet. Barring the consumption of impure products, uridine is unlikely to be harmful. On the other hand, using uridine supplements in unconfirmed subjects may offer false hopes and complicate the interpretation of successful uridine therapy.

## Supplementary information

Supplementary Information
