# Supplementary material for: Cell-based analysis of CAD variants identifies individuals likely to benefit from uridine therapy
Source: Genet Med. 2020 May 28;22(10):1598–605. doi: 10.1038/s41436-020-0833-2 (PMC7521996; doi:10.1038/s41436-020-0833-2)
Supplement: Supplementary file 1 — Supplementary Information [file 41436_2020_833_MOESM1_ESM.docx]

**Supplementary Information**

**Cell-based analysis of *CAD* variants identifies individuals likely to benefit from uridine therapy**

Francisco del Caño-Ochoa, PhD, Bobby G. Ng, BS, Malak Abedalthagafi, MD, Mohammed Almannai, MD, Ronald D. Cohn, MD, Gregory Costain, MD, Orly Elpeleg, MD, Henry Houlden, MD, PhD, Ehsan Ghayoor Karimiani, MD, PhD, Pengfei Liu, PhD, M. Chiara Manzini, PhD, Reza Maroofian, PhD, Michael Muriello, MD, Ali Al-Otaibi, MD, Hema Patel, MD, Edvardson Shimon, MD, V. Reid Sutton, MD, Mehran Beiraghi Toosi, MD, Lynne A. Wolfe, MS, CRNP, BC, Jill A. Rosenfeld, MS, Hudson H. Freeze, PhD and Santiago Ramón-Maiques, PhD

**Table S1.** **Oligonucleotides used for site-directed mutagenesis, CRISPR/Cas9 editing and cloning.**

| **Mutation*^a^*** | **Primer** | **Sequence (5’ – 3’)** |
| --- | --- | --- |
| M33R | *Forward* | TTT CAA ACC GGC AGG GTC GGC TAC CCC GAG |
|  | *Reverse* | CTC GGG GTA GCC GAC CCT GCC GGT TTG AAA |
| Q140R | *Forward* | GGG AAG CTG GTC CGG AAT GGA ACA GAA |
|  | *Reverse* | TTC TGT TCC ATT CCG GAC CAG CTT CCC |
| R238H | *Forward* | GTA TCC ACA CTG AGC CAT GTT TTA TCT GAG C |
|  | *Reverse* | G CTC AGA TAA AAC ATG GCT CAG TGT GGA TAC |
| C252S | *Forward* | CCT GTC TTT GGG ATC AGC CTG GGA CAC CAG CTA |
|  | *Reverse* | TAG CTG GTG TCC CAG GCT GAT CCC AAA GAC AGG |
| I262T | *Forward* | TTG GCC TTA GCC ACC GGG GCC AAG ACT |
|  | *Reverse* | AGT CTT GGC CCC GGT GGC TAA GGC CAA |
| G296E | *Forward* | ACA TCC CAG AAC CAT GAG TTT GCT GTG GAG ACA G |
|  | *Reverse* | C TGT CTC CAC AGC AAA CTC ATG GTT CTG GGA TGT |
| A315T | *Forward* | CTC TTC ACC AAC ACC AAT GAT GGT TCC |
|  | *Reverse* | GGA ACC ATC ATT GGT GTT GGT GAA GAG |
| N320S | *Forward* | AT GAT GGT TCC AGC GAA GGC ATT GTG C |
|  | *Reverse* | G CAC AAT GCC TTC GCT GGA ACC ATC AT |
| G387S | *Forward* | ATT CCC ACT CCC AGC TCT GGA CTT CCA |
|  | *Reverse* | TGG AAG TCC AGA GCT GGG AGT GGG AAT |
| H627N | *Forward* | GAC CCA CTG GGC ATC AAC ACT GGT GAG TCC ATA |
|  | *Reverse* | TAT GGA CTC ACC AGT GTT GAT GCC CAG TGG GTC |
| E682Q | *Forward* | CAG TAT TAC ATC ATT CAG GTG AAT GCC AGG CTC |
|  | *Reverse* | GAG CCT GGC ATT CAC CTG AAT GAT GTA ATA CTG |
| R742Q | *Forward* | GTG GTG AAG ATT CCT CAA TGG GAC CTT AGC AAG |
|  | *Reverse* | CTT GCT AAG GTC CCA TTG AGG AAT CTT CAC CAC |
| D791A | *Forward* | TGT GTG GGC TTT GCC CAC ACA GTG AAA |
|  | *Reverse* | TTT CAC TGT GTG GGC AAA GCC CAC ACA |
| P796T | *Forward* | CAC ACA GTG AAA ACA GTC AGC GAT ATG GAG |
|  | *Reverse* | CTC CAT ATC GCT GAC TGT TTT CAC TGT GTG |
| S995C | *Forward* | TTT GAT GAG ATC TGC TTT GAG GTG GTG |
|  | *Reverse* | CAC CAC CTC AAA GCA GAT CTC ATC AAA |
| V999M | *Forward* | GAG GTG ATG ATG GAC ATC TAT GAG CTC GAG AAC |
|  | *Reverse* | GTC CAT CAT CAC CTC AAA AGA GAT CTC ATC AAA G |
| R1033Q | *Forward* | CGG CAG CAG TGC CAG GTG CTG GGC ACC |
|  | *Reverse* | GGT GCC CAG CAC CTG GCA CTG CTG CCG |
| P1171Q | *Forward* | ACC CCC CAG CAA GAT ATC ACT GCC AAA ACC CTG GAG |
|  | *Reverse* | ATC TTG CTG GGG GGT CAC CAG CGT CGC ATC ACC TG |
| V1217I | *Forward* | GTT ATT GAA TGC AAC ATC CGT GTC TCT CGC TCC |
|  | *Reverse* | GGA GCG AGA GAC ACG GAT GTT GCA TTC AAT AAC |
| G1290S | *Forward* | GGG GAG GTG GCC AGC TTT GGG GAG AGC |
|  | *Reverse* | GCT CTC CCC AAA GCT GGC CAC CTC CCC |
| G1496A | *Forward* | GCC CTG GCT GGG GCC ATC ACC ATG GTG |
|  | *Reverse* | CAC CAT GGT GAT GGC CCC AGC CAG GGC |
| C1501S | *Forward* | ATC ACC ATG GTG AGC GCC ATG CCT AAT |
|  | *Reverse* | ATT AGG CAT GGC GCT CAC CAT GGT GAT |
| A1523V | *Forward* | CTG GCC CAG AAG CTG GTG GAG GCT GGC GCC CGG |
|  | *Reverse* | CCG GGC GCC AGC CTC CAC CAG CTT CTG GGC CAG |
| K1556T | *Forward* | GCA GCC GGG CTG ACC CTT TAC CTC AAT |
|  | *Reverse* | ATT GAG GTA AAG GGT CAG CCC GGC TGC |
| L1557V | *Forward* | GCA GCC GGG CTG AAG GTG TAC CTC AAT GAG ACC |
|  | *Reverse* | GGT CTC ATT GAG GTA CAC CTT CAG CCC GGC TGC |
| E1579K | *Forward* | TG GAG CAT TTC AAG ACA TGG CCC |
|  | *Reverse* | GGG CCA TGT CTT GAA ATG CTC CAT C |
| D1686N | *Forward* | GC TTT GCC TCA AAC CAT GCT CCC CAT ACC TTG G |
|  | *Reverse* | C CAA GGT ATG GGG AGC ATG GTT TGA GGC AAA GC |
| T1716M | *Forward* | CCA CTA CTC CTG ATG GCT GTA AGC GAG |
|  | *Reverse* | CTC GCT TAC AGC CAT CAG GAG TAG TGG |
| R1785C | *Forward* | GGC ACC GTC CGC TGC GTG GTC CTG CGA |
|  | *Reverse* | TCG CAG GAC CAC GCA GCG GAC GGT GCC |
| R1854Q | *Forward* | CAT CTG CCG CCC CAG ATC CAT CGA GCC |
|  | *Reverse* | GGC TCG ATG GAT CTG GGG CGG CAG ATG |
| R1857Q | *Forward* | CCC CGA ATC CAT CAG GCC TCC GAC CCA |
|  | *Reverse* | TGG GTC GGA GGC CTG ATG GAT TCG GGG |
| R1986Q | *Forward* | GCA GCA GCC ATG GCC CAG CTG GGA GGT GCT GTG |
|  | *Reverse* | CAC AGC ACC TCC CAG CTG GGC CAT GGC TGC TGC |
| L1987V | *Forward* | GCA GCC ATG GCC CGG GTG GGA GGT GCT GTG C |
|  | *Reverse* | G CAC AGC ACC TCC CAC CCG GGC CAT GGC TGC |
| R2024Q | *Forward* | GTC GTC GTG CTC CAG CAC CCC CAG CCT GG |
|  | *Reverse* | CC AGG CTG GGG GTG CTG GAG CAC GAC GAC |
| R2110L | *Forward* | GCA CCT CCC AGC CTG CTG ATG CCA CCC ACT GTG |
|  | *Reverse* | CAC AGT GGG TGG CAT CAG CAG GCT GGG AGG TGC |
| E2128K | *Forward* | CGC GGC ACC AAG CAG GAG AAG TTC GAG AGC ATT GAG |
|  | *Reverse* | CTC AAT GCT CTC GAA CTT CTC CTG CTT GGT GCC GCG |
| P2107R | *Forward* | CGC TAC GTG GCA CCT CGC AGC CTG CGC ATG CCA |
|  | *Reverse* | TGG CAT GCG CAG GCT GCG AGG TGC CAC GTA GCG |
| P2186S | *Forward* | ATG CAC CCG ATG AGC CGT GTC AAC GAG |
|  | *Reverse* | CTC GTT GAC ACG GCT CAT CGG GTG CAT |
| **CRISPR*^b^*** | **Primer** | **Sequence (5’ – 3’)** |
| sgRNA1 | *Forward* | caccGGCCGCCGTGTCGACTGCCG |
|  | *Reverse* | aaacCGGCAGTCGACACGGCGGCC |
| sgRNA2 | *Forward* | caccGCGACCCGTCCTCCAACACTA |
|  | *Reverse* | aaacTAGTGTTGGAGGACGGGTCGC |
| sgRNA3 | *Forward* | caccGTTCCCCGGCAGTCGACACGG |
|  | *Reverse* | aaacCCGTGTCGACTGCCGGGGAAC |
| **cloning*^c^*** | **Primer** | **Sequence (5’ – 3’)** |
| huCAD | *Forward* | agcacagtggcggccgcATGGCGGCCCTAGTGTTG |
|  | *Reverse* | aaacgggccctctagactagAAACGGCCCAGCAC |
| exon1 | *Forward* | TTCCAGTGGAGTTTGCAGTC |
|  | *Reverse* | CTTGCAGAGACCGAACTCAT |

*^a^* Nucleotides in red introduce clinical variant.

*^b^* Lower case sequence is complementary with restriction site.

*^c^* Lower case sequence indicates region for In-Fusion cloning.
